# Low‐dose spinal and opioid‐free anesthesia in patient with Severe Aortic Stenosis and SARS‐CoV‐2 infection: Case report

**DOI:** 10.1002/ccr3.4192

**Published:** 2021-08-21

**Authors:** Antonio Coviello, Marilena Ianniello, Ezio Spasari, Concetta Posillipo, Maria Vargas, Alfredo Maresca, Giuseppe Servillo

**Affiliations:** ^1^ Department of Anesthesiology and Intensive Care Medicine Policlinico ‐ Federico II University Hospital Naples Italy

**Keywords:** aortic stenosis, combined spinal‐epidural anesthesia, COVID‐19, SARS‐CoV‐2, total knee replacement

## Abstract

The best anesthesiologic approach to severe AS patient has not been adequately studied in literature. Although the current guidelines have a cautious attitude in this regard, Combined Spinal‐Epidural Anesthesia (CSEA) has proved to be a safe technique. Therefore, we would like to provide our experience with a severe AS and COVID‐19 patient.

## Graphical abstract

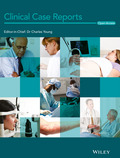

## 1 INTRODUCTION

An 81‐year‐old patient with Severe Aortic Stenosis (AS) and SARS‐CoV‐2 infection came for a Total Knee Replacement (TKR) due to periprosthetic joint infection (PJI). After reviewing the disease‐related factors, we decided to perform a combined spinal‐epidural anesthesia (CSEA) in order to maintain safety standards for patient and healthcare workers.

SARS‐CoV‐2 infection is responsible for various manifestations ranging from asymptomatic infection to severe viral pneumonia with respiratory failure.^1^ Measures to minimize the virus spread, exposure, and transmission are vital, especially for healthcare workers and patients. Aerosol‐Generating Procedures—such as tracheal intubation—should be avoided in positive pressure room (such as operating rooms) due to the risk of airborne transmission.^2^ Therefore, neuraxial and regional anesthesia may be safe anesthetic care choices with a lower risk of virus exposure and postoperative complications. Severe AS is an intraoperative management challenge for anesthesiologists, due to the risk of hypotension, decreased coronary perfusion pressure, cerebral hypoperfusion, cardiac failure and sudden death.^3^ Therefore, American Heart Association (AHA) and European Society of Cardiology (ESC) guidelines on perioperative cardiovascular evaluation and care for noncardiac surgery recommend General Anesthesia (GA) to ensure a better hemodynamic stability, considering Neuraxial Anesthesia (NA) relatively contraindicated.^4^ The reviewed literature consistently shows positive results and good hemodynamic stability with NA management in severe AS patients.^5^ Furthermore, NA allows to perform an opioid‐free anesthesia (OFA) reducing the risk of opioid‐induced respiratory depression (OIRD)^6^ and to avoid GA‐correlated induction‐triggered hypotension, wide fluctuation in hemodynamics, pulmonary complications, and cardiac dysrhythmias.^7^ In this regard, during this healthcare crisis, considering the French Society of Anaesthesia and Intensive Care (SFAR)^8^ recommendations and our COVID‐19 patient conditions, we established to carry out a NA in order to limit the risk of contamination to patients and healthcare workers.

## 2 CASE HISTORY

An 81‐year‐old female patient was referred to our Department of Surgical Sciences, Orthopedic Trauma and Emergencies (University of Naples “Federico II”) to replace her infected left knee prosthesis. The prosthesis was exposed, the surgery involved replacement of the prosthesis and plastic reconstruction of tissues.

### 2.1 Preoperative evaluation

At the preoperative anesthesiologic examination, the patient reported COVID‐19 infection, chronic obstructive pulmonary disease (COPD), hypertension, iron‐deficiency anemia, venous insufficiency, severe chronic kidney disease (CKD—stage IV) and severe AS. She reported a severe limitation of physical activity tolerance (NYHA 3), body mass index (BMI) 29.1 kg/m^2^, cough, oxygen‐therapy at a Fraction of inspired Oxygen (FiO_2_) of 35% and a difficult weaning on a previous general anesthesia for uncomplicated Total Knee Replacement (TKR) implantation. The preoperative ECG showed bradycardia, right bundle branch block (RBBB), and junctional rhythm. Chest X‐ray showed increased bronchovascular markings and cardiomegaly. Spirometry and arterial blood gas (AGB), performed during the anesthesiologic evaluation, and underlined a moderate airflow obstruction and hypercapnic hypoxia. Further cardiological study was required according to the European Society of Cardiology (ESC) 2019 guidelines—Revised Cardiac Risk Index^9^ (RCRI—class III—10.1% 30‐day risk of death, miocardial infarction or cardiac arrest); Acute Physiology and Chronic Health Disease Classification System II (APACHE II) score 14 points (7% estimated postoperative mortality, 15% estimated nonoperative mortality); Physiological and Operative Severity Score for the enUmeration of Mortality and Morbidity (POSSUM) score (63.2% predicted mortality, 96.6% predicted morbidity); Surgical Outcome Risk Tool (SORT) score (risk 4.72%). A two‐dimensional (2‐D) echocardiography showed severe AS (aortic valve area = 0.75 cm^2^, pressure gradient = 78/44 mmHg; ejection fraction = 45%) and concentric left ventricular (LV) hypertrophy. Considering the condition of AS, the cardiac surgery should have been carried out first, but the patient refused cardiac surgery because of her concern relating to cardiac surgery.

### 2.2 Anesthesia and intraoperative management

In multidisciplinary agreement with team of cardiologists, orthopedics, and plastic surgeons, and in agreement with the patient herself, it was decided the anesthesiologic technique and we chose CSEA, which was considered to be the safest anesthetic approach, to use considering the impact of GA on patient's cardiac and pulmonary conditions and a recovery in Surgical Intensive Care Unit (SICU) was disposed. On the day of surgery, antibiotic prophylaxis was administered (Vancomycin 15 mg/kg with slow intravenous infusion) 60 minutes before skin incision. Pulse oximetry (SpO_2_), heart rate (HR), body temperature (C°), continuous invasive arterial (cIBP), central venous pressures (CVP), cerebral oximetry with ForeSight were monitored. Elastic stockings were applied to both legs to minimize blood pooling and to reduce spinal hypotension. A coloading was started with 500 mL of intravenous crystalloids and a Venturi mask at FiO_2_ 35% was applied. In asepsis, combined Spinal‐Epidural Anesthesia (CSEA) was performed with the patient in the left lateral decubitus position after determining the L3‐L4 interspace with anatomical method, identifying L4 with the bisiliac line. The level of puncture was confirmed by ultrasound counting the vertebrae from the sacrum, in a caudo‐cranial sense. A needle‐in‐needle technique was used with spinal needle 27 Gauge Whitacre point and with epidural needle Thuoy 17 Gauge. The epidural space was identified at 5 cm from the skin through the loss of resistance in the water spindle (prefilled syringe of sterile saline solution). Bupivacaine 0.25% 5 mg combined with ketamine 0.1 mg/kg and midazolam 1 mg were injected into the subarachnoid space. The peridural catheter was inserted 5 cm in the epidural space (10 cm from the skin). Negative pressure occurred in the catheter and negative aspiration for blood and Cerebrospinal fluid (CSF). Epidural test dose was performed according to recommendations, injecting epinephrine 15 micrograms through the epidural catheter and waiting for 5 minutes to check for any cardiovascular symptoms. The test was negative. The patient was then immediately turned to a supine position. After 5 minutes, the anesthesiologic plane was tested through pin‐prick test and ice test, assessing the extension of the sensory block between T12‐S4 with Hollmen grade 4 (loss of sensation). Motor block was rated as Bromage I (the patient was unable to move her feet or knees). Patient's vital signs after CSEA were stable. Serial arterial blood gases (ABGs) were performed hourly to monitor her pH, oxygen (O2), carbon dioxide (CO2), and lactate that were unvaried during surgery. Throughout the surgery 0.375% ropivacaine plus dexamethasone 8 mg (total volume 20 mL), divided in boli (5 mL per bolus), was administered in separate doses through the epidural catheter, titrated on patient's hemodynamics and anesthetic plane. Blood loss was minimal (about 500 mL). None hemodynamic instability event occurred (IBP average about 70 mm Hg, HR about 60 bpm and SpO_2_ about 93%). The total duration of surgery was about 8 hours. At the end of the surgery, Bromage Motor Blockade Score and Hollmen score were evaluated, respectively, as grade 4 and grade 2.

### 2.3 Postoperative management

We discharged her with a Programmed intermittent epidural boluses / patient controlled epidural analgesia (PIEB / PCEA) epidural pump with programmed boluses of Ropivacaine 0,1% (8 mL per hour) and the possibility of boluses at the patient's request (5 mL per hour, with safety lock for the next 60 minutes). Thereafter, the patient was transferred in our surgical intensive care unit (SICU) without any hemodynamic instability or cardiovascular/neurologic complications, where her vital signs, hourly diuresis and cardiac enzymes, were monitored for the next 48 hours and every six hours a Visual analog scale for pain (VAS) was administered. We carried out a serial check of the patient, monitoring the appearance of any side effects such as nausea or vomiting, pruritus, shivering. In the first day after surgery, the patient used the PCEA twice, administering the additional ropivacaine bolus. The patient did not use the PCEA on the second day. Pain control was optimal, with VAS = 2 on the first day after surgery, VAS = 0 from the second day. There were no side effects.

## 3 DISCUSSION

Neuraxial Anesthesia (NA) is relatively contraindicated in patients with severe AS, due to the significant risk of perioperative morbidity and mortality. NA’s sympatholytic effect may cause loss of vascular tone and reduction of cardiac output, resulting in hypotension and decreased coronary perfusion pressure and these changes can cause significant hemodynamic compromise in AS patients with diminished cardiac reserve. However, GA is involved in multiple complications such as induction‐triggered hypotension, wide fluctuation in hemodynamics, pulmonary complications, and cardiac dysrhythmias,^10^ as well as the levels of stress hormones, are well‐documented by Bundgaard‐Nielsen et al^7^ and Guay J.^11^ Furthermore, a meta‐analysis performed by Guay et al^12^ underlined that NA alone, compared with GA (with or without supplementary NA), significantly reduced the mortality rate by 2.5% and the risk of perioperative pneumonia. On this regard, White et al showed the inverse correlation between hypotension, mortality, and a minimal dose of intrathecal local anesthetic in fragile patients.^13^ This technique has been demonstrated to be safe especially for patients with stenotic cardiac disease, also by Ben‐David et al, underlining the lower need for vasopressors in the low‐dose spinal group versus the standard‐dose group.^14^ Considering these findings, we performed a combined spinal‐epidural anesthesia gradually administrating a low dose of local anesthetic using ketamine as an adjuvant to bupivacaine, in attempt to avoid mean arterial pressure (MAP) fluctuations.

### 3.1 Ketamine‐ and opioid‐free anesthesia

Ketamine is an N‐methyl‐D‐aspartate (NMDA) receptor antagonist with both central and peripheral analgesic effects that has been used as intrathecal adjuvant to local anesthetic due to its sedative and analgesic effects, contributing to the maintenance of hemodynamic stability and minimizing stress‐induced perioperative disorders. Intrathecal ketamine significantly reduce postoperative IL‐6 and c‐reactive protein (CRP) inflammatory response, through inhibition of nuclear factor (NF)‐KB, which is involved in proinflammatory cytokines production.^15^ Camila A. Carpi et al in their study observed that intrathecal Ketamine had the intraoperative hemodynamic stability, level of blockage, and other secondary outcomes comparable to the morphine with lower postoperative incidence of pruritus.^16^

The effect of the intrathecal ketamine with spinal bupivacaine on the onset of sensory block is not clear. These apparently controversial results may be due to the different populations, doses of ketamine, and methodologies. Marzieh Beigom Khezri et al speculate that the pH of the solution is a possible reason why ketamine prolongs the onset of sensory block. The pH of ketamine hydrochloride is slightly acidic (3.5e5.5).^17^ Results of the clinical study by Galindo suggested that the pH‐adjusted solutions of local anesthetics produced a more rapid onset of blockade with better quality and longer duration than the unmodified commercial preparations.^18^ Moreover, Ritchie et al confirmed that the uncharged molecule is essential for penetration to the intracellular receptor site. The addition of ketamine decreases the pH of bupivacaine, and therefore, the onset of the sensory block is prolonged.^19^ Bion et al declared that the use of intrathecal ketamine was associated with minimal hemodynamic fluctuations. They reported that the transmission of ketamine into the venous system (azygos vein) of the spinal cord‐induced cardiovascular stimulation and hemodynamic stability after spinal anesthesia.^20^

On the other hand, CSEA allows to perform an opioid‐free anesthesia (OFA) for reducing the risk of opioid‐induced respiratory depression (OIRD) and minimizing postoperative nausea and vomiting (PONV) and constipation, allowing for an earlier mobilization.^6^

Furthermore, opioids suppress effector functions of both innate and adaptive immune system, reducing phagocytosis and enhancing viral replication, not desirable in a COVID‐19 patient. Besides, the use of epidural dexamethasone was chosen not only for its property of reducing the total amount of bupivacaine requirement and prolonging epidural anesthesia duration^21^ but also because of decreasing mortality in patients with SARS‐CoV‐2 infection who were receiving oxygen without invasive mechanical ventilation.^22^

### 3.2 Anesthesiologic choice and management

Combined Spinal‐Epidural Anesthesia (CSEA) has been demonstrated to have more advantages, compared to epidural anesthesia alone, considering the leakage of epidural local anesthetic through the dural hole in the subarachnoid space and the perineural or transdural spread of epidural local anesthetic to higher metamers.^23^ Starting from these considerations, we preferred to perform CSEA in a patient with AS to make TKR and reconstructive surgery. Our main goals were to minimize hemodynamic changes, to avoid acute kidney injury (considering our patient's CKD), to decline the risk of pulmonary complications and, considering this COVID‐19 pandemic, to minimize the virus spread, exposure, and transmission (avoiding the use of Aerosol‐Generating Procedures, such as tracheal intubation).^2^ We performed epidural test dose with epinephrine 15 micrograms to rule out accidental placement in the subarachnoid space or in a blood vessel, as recommended by scientific literature. Therefore, Continuous Spinal Anaesthesia (CSA) may be more interesting in patients with severe AS, considering the possibility of titrating gradually local anesthetic, using smaller doses, and leading to maintenance of hemodynamic stability, as documented by Minville et al This technique is also easier and safer than CSEA, due to the certainty that catheter is well‐positioned through aspiration of CSF; the same certainty is not guaranteed using an epidural catheter. However, the lack of adequate spinal set greatly limits the use of this technique, considering the frequency of spinal catheter structural damages.^24^ A postoperative pain management with PIEB/PCEA was applied because it provides better pain relief with less local anesthetic consumption with lower cardio or neurotoxicity and less motor block, compared with continuous epidural infusion (CEI), allowing a faster recovery. We could practice lumbar plexus block in order to avoid any risk of dural puncture. Bin Mei et al used this technique during hip surgery when it was preferable to avoid neuroaxial approach.^25^ In our case we risked to not obtain a long‐lasting anesthesiologic coverage, also we would not obtain adequate coverage of postoperative pain without using opioids, which would increase the risk of respiratory depression. Although a combined sciatic/femoral and lateral cutaneous nerve block for TKR could be performed in this case to minimize the risk of hemodynamic fluctuations related to NA, the risk of local anesthetic toxicity would be effectively increased considering the total volume (15‐20 mL, 10‐15 mL and 10 mL for sciatic, femoral, and lateral cutaneous nerve block, respectively), and dose administration. Our report illustrates the use of CSEA with minimally invasive hemodynamic monitoring as a valid alternative to general or spinal anesthesia in a patient with severe aortic stenosis and SARS‐CoV‐2 who is undergoing knee surgery. However, controlled‐clinical trials would be required to establish that this technique is safe and effective in these type of patients.

## 4 CONCLUSIONS

During the intraoperative and postoperative period, our severe AS and COVID‐19 patient presented hemodynamic stability, good respiratory compensation, and optimal pain control VAS = 0. Although ACC/AHA guidelines not recommend the use of a NA in symptomatic severe AS patients, this report shows that the anesthesiologic management should be applied to the context, considering severity of AS symptoms. In conclusion, the successful management of this case suggests that CSEA can be considered as an effective safe choice in severe AS and COVID‐19 patients, considering the risks/benefits balance. Although, we are sure that the continuous spinal anesthesia (CSA) would be the gold standard in patients with respiratory and cardiovascular diseases, after solving CSA’s set structural lack and difficult postoperative management.

## CONFLICT OF INTEREST

None declared.

## AUTHOR CONTRIBUTIONS

All persons who meet authorship criteria are listed as authors, and all authors certify that they have participated sufficiently in the work to take public responsibility for the content, including participation in the concept, design, analysis, writing, or revision of the manuscript. Furthermore, each author certifies that this material or similar material has not been and will not be submitted to or published in any other publication before its appearance in this journal. Antonio Coviello, Maria Vargas, Ezio Spasari: conceptualized and designed the study. Antonio Coviello, Concetta Posillipo, Ezio Spasari: acquired the data. Antonio Coviello, Marilena Ianniello, Alfredo Maresca: analyzed and interpreted the data. Marilena Ianniello, Concetta Posillipo, Ezio Spasari: drafted the manuscript. Antonio Coviello, Maria Vargas, Alfredo Maresca: revised the manuscript critically for important intellectual content. Antonio Coviello, Alfredo Maresca, Giuseppe Servillo: approved the version of manuscript to be published.

## CONSENT FOR PUBLICATION

Written informed consent was obtained from the patient for publication of this case report and its accompanying image. A copy of the written consent is available for review by the Editor‐in‐Chief of this journal.

## Data Availability

The authors confirm that the data supporting the findings of this study are available within the article and its supplementary materials.
